# Exploring Early Micronutrient Deficiencies in Rainbow Trout (*Oncorhynchus mykiss*) by Next-Generation Sequencing Technology – From Black Box to Functional Genomics

**DOI:** 10.1371/journal.pone.0069461

**Published:** 2013-07-24

**Authors:** Pål A. Olsvik, Gro-Ingunn Hemre, Rune Waagbø

**Affiliations:** National Institute of Nutrition and Seafood Research (NIFES), Bergen, Norway; Temasek Life Sciences Laboratory, Singapore

## Abstract

This work studies final nutritional status and transcriptional responses of rainbow trout (*Oncorhynchus mykiss* Walbaum 1792) (28 g) after a 10 week feeding experiment designed to elucidate the effect of adding a vitamin and mineral premix on growth, health, and nutritional endpoints. Juvenile fish were fed a either a diet supplemented with a vitamin and mineral premix (Diet S) or the same diet without premix supplementation (Diet U). The analyzed micronutrient composition of diets differed accordingly. Pooled livers from 15 fish from each dietary group were used to create suppression subtractive hybridization (SSH) cDNA libraries that were sequenced with 454 FLX GS Titanium Technology. In total 552 812 reads were sequenced from the two cDNA libraries. Ingenuity pathway analysis (IPA) was then used to characterize the hepatic transcriptome of the two dietary groups of rainbow trout. In the present communication we discuss how selected micronutrients may affect the transcriptome at suboptimal status by directly impacting the cellular metabolism, functions, and structures, and by introducing respective compensatory mechanisms. Processes related to lipid metabolism, peptide hydrolysis, oxygen transportation, and growth development were mostly affected. Considering the transcriptomics data relative to changes in nutritional status from the feeding study and the background phenotypic outcome of growth performance and gill histopathology, the outcome of the transcriptional profiling are suggested to be mainly related to suboptimal pantothenic acid and vitamin C nutrition.

## Introduction

It is generally acknowledged that the limited global fish meal and fish oil production cannot support the future growth in aquaculture, so there is a need for large and stable supplies of suitable alternatives, like selected plant proteins and lipids [1], [2]. High fishmeal and fish oil replacement levels have successfully been adopted, however with increasing concern that the modern diets do not fulfill the requirements of all essential nutrients. It is therefore a constant need for more and better knowledge on nutritional requirements for farmed fish. Traditional feed formulations with use of fishmeal (proteins) and oils (lipids) contained enough of most micronutrients needed by the fish. To ensure adequate intakes of these, additional levels of selected components are added to feeds through micronutrient premixes. Vitamin and mineral premixes are concentrates of stable forms of essential vitamins mixed with essential elements in carriers, added to feeds separately. Vitamin premixes for farmed salmonids typically contains vitamin E, vitamin C, vitamin K_3_ and the eight B-vitamins [2], [3]. Mineral premixes are concentrates of essential elements, often added in surplus levels to ensure that potential antagonistic interactions among feed ingredients are avoided. Among the supplemented trace elements in salmonids diets are manganese, iodine, copper, zinc and iron [2], [4].

Present recommendations on vitamin and mineral requirements are based on minimum requirements established from feeding experiments with semi synthetic diets (semi purified ingredients) or practical diets made on high quality marine ingredients [2]. Challenges when modern diets are replacing parts of the fish meal protein and marine lipids with plant ingredients are changes in concentrations of inherent micronutrients, changes in their chemical forms, as well as including compounds interacting with micronutrient uptake and metabolism. The nutritional history includes a lot of examples of single nutrient deficiencies that have caused reduced production and health in farmed fish [5]. Indeed, many micronutrient deficiencies have been seen after changes in feed ingredients [6]. To allow for flexibility with respect to use of new and sustainable feed ingredients the fish feed industry increase their focus on the micronutrient content and bioavailability, and the need for supplementation. In this work, both classical and new tools for detecting micronutrient deficiencies are needed, preferably sensitive early indicators.

Transcriptomic profiling has become a useful nutrigenomic discovery tool for identifying the molecular basis of biological functions underlying responses to nutrition and in search for novel nutritional biomarkers in fish [7], especially focusing nutritional immunology [8] and exploring functional health promoting feeds [9]. Even though microarrays remain the primary technology for transcriptomic profiling, an emerging alternative is to use next-generation sequencing to directly sequence and quantify transcripts from experimental samples. Compared to microarrays, next-generation sequencing technologies offer several advantages in functional genomics research, including a much wider dynamic range of detection [10]. Next-generation sequencing is especially promising for functional genomic research in non-model species, for which genomic data are underdeveloped.

Explorative nutrigenomic studies with focus on micronutrients are missing [7]. The present report is based on biological material from a ten-week feeding experiment with rainbow trout (*Oncorhynchus mykiss*) designed to elucidate the preventive effect of adding a vitamin and mineral premix (supplementary diet – Diet S) in practical feeds (equal ration of plant and marine ingredients) for juvenile rainbow trout on growth, nutritional endpoints and on hepatic transcriptional responses. Fish fed an unsupplementary diet (Diet U), with borderline micronutrient status were used as reference. Pooled livers from 15 fish fed Diet S and 15 fish fed Diet U were used to create suppression subtractive hybridization (SSH) cDNA libraries that were sequenced with 454 FLX GS Titanium Technology. Ingenuity pathway analysis (IPA) was then used to characterize the hepatic transcriptome of the two dietary groups of rainbow trout. Following an exploring approach it was of interest to see if differences in the liver transcriptome among the dietary groups fitted the described phenotypic symptoms of suboptimal nutrition or deficiencies observed in the feeding experiment (growth depression, nutrient status and initial gill pathology) in fish fed the unsupplemented diet. The identified related genes could be useful as markers in micronutrient studies.

## Materials and Methods

### 2.1. Animal trial and experimental feeds

The present biological material originated from a feeding trial conducted in Denmark by Biomar, Denmark. These studies do not require special approval from the national animal research agency (Committee for Animal Experimentation) according to Danish legislation. Biomar, Denmark research facility has an approval to raise farmed fish according to the governmental central husbandry register (Statens centrale husdyrbrugsregister (CHR registret), CHR-nummer 103755), CHR-register number 103755. The fish were anaesthetized with 50 mg/L tricaine methanesulfonate (MS-222) and were sacrificed by terminal anaesthetization with tricaine methanesulfonate (MS-222) (200 mg/l) followed by a blow to the head.

The present experiment was part of a comprehensive study examining need for vitamin and mineral supplements in an extruded feed for rainbow trout based on 50∶50 marine and plant ingredients [11]. While the complete experiment covered groups of rainbow trout fed gradually increased vitamin and mineral premix designed to cover 0, 25, 50, 100 and 200% supplementation based on NRC [2] recommendations. This communication cover a comparison of fish groups fed the 100% premix diet (supplemented Diet S) and the unsupplemented diet (Diet U). Healthy rainbow trout (27.7 g±4.3 g) of same genetic and environmental background were randomly distributed into six indoor tanks (0.4 m^3^), each stocked with 100 fish. The fish were acclimatized to the tank conditions one week prior to the start of the trial. The experiment was conducted in recirculated freshwater at stable light (LD12∶12) and temperature (16°C±1°C) conditions, while water quality parameters like water oxygen levels, conductivity and pH recorded regularly were within acceptable normal levels.

A basal diet mixture was used for the two high-energy extruded feeds which were produced at BioMar TechCenter (Brande, Denmark) as 2 and 3 mm pellet sizes. The same basal feed recipe contained equal amounts of marine and vegetable protein and oil raw materials, and commercial levels of binder, antioxidants and inorganic phosphorus (Table 1). The two experimental feeds were obtained by adding a defined vitamin and mineral premix to the basal feed recipe at 3 g kg^−1^ (supplemented diet – Diet S) or using the basal feed recipe unsupplemented premix (Diet U). Consequently the feeds differed in vitamin E, vitamin K_3_, thiamin (B_1_), riboflavin (B_2_), niacin, pantothenic acid, pyridoxine (B_6_), biotin, folic acid, B_12_ and vitamin C) and minerals zinc (Zn), iodine (I), copper (Cu), cobalt (Co) and manganese (Mn) concentrations. Table 1 shows the experimental basal feed recipe and macro nutrient composition, while Table 2 shows supplementations of vitamins and minerals from the premix in Diet S and the analyzed micronutrient concentrations in Diets U and S. Each feed was provided to triplicate tanks in two meals per day.

**Table 1:** The experimental basal feed recipe (g kg^−1^) and macronutrient composition in the experimental diets.

Ingredients	g kg^−1^
Fishmeal	500
Binder	132
Vegetable protein	278
Fish oil	106
Vegetable oil	106
Vitamin and mineral premix	0 or 6
Additives	10

Proximate composition	
Protein	452
Fat	264
Ash	92
Water	70
*Amino acids*	
Histidine	12.4
Lysine	31.7
Methionine	11.7

**Table 2:** Target supplementations of vitamins and minerals (mg kg^−1^) from the premix in the two experimental diets (unsupplemented Diet U and supplemented Diet S), respective analyzed feed values, present nutrient requirements from the NRC (2011), and significant changes in respective tissue status between the dietary groups.

	Supplemented		Analyzed					
Micronutrient Method	Diet U	Diet S	Diet U	Diet S	NRC 2011*)	Analyzed tissue	Difference in body status^**)^	
***Vitamins***								
Vitamin E [49]	0	100	44	103	50	Liver	3.9	
Vitamin K_3_ (MD) [50]	0	10	0.2	1.2	R	Liver	ns^***)^	
Thiamin (B_1_) [51]	0	10	3	10	1	Muscle	ns	
Riboflavin (B_2_) [52]	0	12	5	14	4	Muscle	ns	
Niacin [53]	0	30	54	86	10	Muscle	ns	
Pantothenic acid [53]	0	40	6	36	20	Muscle	3.3	
Pyridoxine (B_6_) [41], [54]	0	10	3/35	8/153	3	Muscle	2.9/4.4^****)^	
Biotin [53]	0	0.3	0.3	0.5	0.15	Muscle	ns	
Folic acid [53]	0	10	1	6	1	Liver	1.4	
Vitamin B_12_ [53]	0	0.01	0.09	0.09	R	Muscle	ns	
Vitamin C [55	0	70	0	57	20	Liver	8.5	
***Elements***								
Zinc (Zn) [56]	0	100	60	155	15	Whole body	1.6	
Iodine (I) [57]	0	0.6	0.9	1.5	1.1	Whole body	ns	
Copper (Cu) [56]	0	1	5	6	3	Whole body	ns	
Cobolt (Co) [56]	0	1	0.2	1.2	?	Whole body	ns	
Manganese (Mn) [56]	0	8	29	36	12	Whole body	ns	

*) For rainbow trout; R: required;? not determined; ^**)^ Significant differences in organ status in rainbow trout fed Diets S relative to U (p<0.05) given as status Diet S/status Diet U; ^***)^ns not significant difference; ^****)^ Muscle ASAT activity (U/g protein).

The micronutrient analyses in the feed were analyzed according to the methods referred to in Table 2. Retention of single micronutrients added to the diet S were calculated as: % of added  =  [(analyzed in Diet S – analyzed in Diet D) *100] supplemented in Diet S^−1^.

### 2.2. Sampling, biological performance data and micronutrient status analyses

Periodical specific growth rate (% SGR) was calculated as % daily growth increase during the 5 initial and 5 final weeks: SGR  =  (ln BW_2_ – ln BW_1_ * days of experiment^1^) * 100; where BW_1_ and BW_2_ represent the initial and final body weights in grams, respectively. Further information on biological performance of the present study refers to growth, feed digestibility and nutrient retention that is reported elsewhere [11] or given as own results not shown.

Sampling was conducted at the completion of the feeding trial. The fish were starved for 48 hours before sampling. Sampled fish were sedated with 50 mg l^−1^ MS-222 and anaesthetized with 200 mg l^−1^ MS-222. The fish were killed by a sharp blow to the head. White dorsal muscle and liver tissue and whole fish from the final sampling after 10 week were used for micronutrient status analysis, while liver tissue was used for sequencing and qPCR analysis. Each tank was randomly sampled for 10 fish by netting after lowering the water volume, of which 5 fish was used for whole body analyses. For the other five fish, liver and white muscle tissues were dissected. Individual liver samples were used for sequencing and qPCR, while pools of liver and white muscle tissues from these fishes were used for the vitamin and mineral status analyses. Tank triplication gave 15 individual liver samples and 3 respective pooled samples per dietary group. Whole fish were dip frozen in liquid nitrogen. Similarly, pooled organ samples per tank were immediately frozen on liquid nitrogen or on dry ice, while individual muscle and liver samples for molecular analyses were flash frozen in liquid nitrogen. All samples were kept frozen on −80°C until analysis. Gill tissue (2^nd^ gill arch) was placed on buffered formalin for histological examination, and gill histology were performed according to standardized preparation, staining and evaluation by the Norwegian Veterinary Institute (Bergen, Norway).

All micronutrient status analyses were performed on pooled samples (each of 5 fish) per tank (n = 3 per dietary group) of whole body, liver and muscle homogenates of rainbow trout fed Diets S and U. The target and storage organs for micronutrients differ and organ analyses were therefore chosen according to their respective suitability to reflect status in requirement studies (summarized in NRC 2011). Some nutrients were also prioritized according to practical available amount of liver tissue. Respective status organ and method of analysis for the micronutrients are listed in Table 2. Differences in status of single micronutrients between fish fed the Diets S (Group S) and U (Group U) were calculated as % increased status in fish fed Diet S relative to Diet U: % increase  =  [(status in Group S – status in Group U) *100] status Group U^−1^.

### 2.3. RNA isolation

Liver tissues from 15 individual rainbow trout per dietary group from the final sampling were thoroughly homogenized before RNA extraction using a Precellys 24 homogenizer by ceramic beads CK28 (Bertin Technologies, Montigny-le-Bretonneux, France). Total RNA was extracted using the BioRobot EZ1 and RNA Tissue Mini Kit (Qiagen, Hilden, Germany) and treated with DNase according to the manufacturer's instructions and eluted in 50 μl RNase-free MilliQ H_2_O. The RNA was then stored at –80°C before further processing. RNA quality and integrity were assessed with the NanoDrop ND-1000 UV-Vis Spectrophotometer (NanoDrop Technologies, Wilmington, DE, USA) and the Agilent 2100 Bioanalyzer (Agilent Technologies, Palo Alto, CA, USA). The RNA 6000 Nano LabChip kit (Agilent Technologies, Palo Alto, CA, USA) was used to evaluate the RNA integrity of the liver samples. The 260/280 and 260/230 nm ratios of the extracted RNA were 2.1±0.0 and 2.0±0.1 (n = 30), respectively (mean ± SD). The RNA integrity number (RIN) of the liver samples used for RT-qPCR was 9.0±0.2 (n = 30) (mean ± SD).

### 2.4. Suppressive subtractive hybridization (SSH) library construction and 454 FLX sequencing

Pooled RNA from 15 livers of rainbow trout from each of the two treatment groups was used to construct cDNA for sequencing. mRNA from each sample was isolated using the NucleoTrap mRNA Mini Kit (Macherey-Nagel, Düren, Germany). The Agilent Bioanalyzer with the RNA 6000 Nano LabChip kit and the DNA 7500 Kit (Agilent Technologies, Waldbronn, Germany) was used to evaluate the quality of the mRNA and cDNA samples used for cDNA library construction. 200 ng of mRNA from each sample was pooled for cDNA synthesis according to the GS FLX Titanium Rapid Library Preparation Kit (Roche Applied Sciences, Basel, Switzerland).

Suppressive subtractive hybridization (SSH) was performed using the Clontech PCR Select cDNA Subtraction Kit (Clontech, Mountain View, CA) following the manufacturer's recommendations. cDNA subtraction was performed in both directions. Forward subtracted libraries were designed to be enriched for genes that were up-regulated in liver of rainbow trout by the unsupplemented diet (Diet U), and reverse subtracted libraries were designed to be enriched for genes that were down-regulated by Diet U. Pooled Diet U mRNA samples from 15 rainbow trout livers were used as testers in the forward subtractions and as drivers in the reverse subtractions. Pooled supplemented diet (Diet S) fish mRNA samples from 15 livers were used as drivers in the forward subtractions and as testers in the reverse subtractions. To evaluate subtraction efficiency, the abundance of transcripts of the housekeeping gene ubiquitin was examined by PCR.

Liver tissue cDNA libraries from rainbow trout were prepared as stated above and sequenced according to the Roche protocol using 454 GS FLX (Titanium chemistry) at the Ultra-high Throughput Sequencing Platform of the Centre for Ecological and Evolutionary Synthesis (CEES), Department of Biology, University of Oslo. Nucleotide sequences were incorporated into two SFF files using the 454's software and applied in downstream analyses.

### 2.5. Clustering, assembly and read processing

As a quality measure in search for possible microbial contamination, i.e. impurities in the nucleotides under investigation, all reads generated by the FLX sequencer were subjected to taxonomic profiling using MEtaGenome ANalyzer (MEGAN, version 3.9.) [12], using default settings. Reads longer than 100 nt were aligned to the GenBank non-redundant protein database (BLASTX) [13] using a cut-off e-value of 1e-6, at the University of Oslo Bioportal (www.bioportal.uio.no), and the BLAST results used as input in the MEGAN analyses.

Quality filtered flowgram (SFF) files were assembled using the GS De novo Assembler v2.6 (Newbler) software (Roche Diagnostic) with minimum overlap 80 bp and minimum identity 96% (“-ml 80 -mi 96 -cdna –ace”). Two sets of assembled “isotigs” (contigs) were used in the downstream functional analyses. For gene ontology (GO) analyses, all isotigs consisting of 6 or more reads were used, whereas for Ingenuity Pathway Analysis (IPA) analyses, isotigs consisting of more than 50 reads were used. From the Diet U group there were 1 789 isotigs with >6 reads and 662 isotigs with >50 reads, while from the Diet S group there were 4 350 isotigs with >6 reads and 636 isotigs with >50 reads. Blast2GO [14] was used to annotate each isotig from the two SSH cDNA library assemblies. Blast2GO analyses were run using a cut-off e-value of 1e-3 (BLASTX) and 1e-6 (mapping). GO enrichment analyses were performed with Fisher's exact test applying the GOSSIP tool [15] as integrated in the Blast2GO software.

For the IPA analyses, isotigs with more than 50 reads from the forward and reverse SSH cDNA libraries were used (in total 1 297 sequences). All assembled isotigs with positive annotation from the Diet U fish group, representing genes higher expressed in liver of rainbow trout fed the unsupplemented diet compared to the supplemented diet, were given a positive score of 2, whereas assembled isotigs with positive annotation in the Diet S fish group, representing genes lower expressed in the fish fed the unsupplemented diet, were given a negative score of −2. The resulting gene list, with genes either up- or down-regulated in liver of rainbow trout from the Diet U group, was used as input for functional analysis via IPA. Since IPA only can map mammalian homolog identifiers, GeneCards IDs were submitted for biological function and pathway analysis, using top BLASTX hits and assuming orthologous genes have the same function. IPA could map and identify 834 differently expressed genes of the 1 297 annotated isotigs included in the gene list.

### 2.6. Quantitative real-time RT-qPCR

PCR primer sequences used for quantification of the transcriptional levels of selected genes, as well as the reference genes, are shown in Table 3. In total 20 genes were quantified with RT-qPCR, of which 3 were selected as potential reference genes. BLASTX or BLASTN was used to determine PCR assay specificity. The reaction specificity of each assay was checked by examining the melting curves generated with a dissociation protocol from 65 to 97°C.

**Table 3:** PCR primers, accession numbers, amplicon sizes and PCR efficiencies.

Gene	Accession no.	Forward primer	Reverse primer	Amplicon size (bp)	PCR efficiency
*cyp1a3*	AF015660	GTGAGGCCATCGGACACAAC	GACAGCGCTTGTGCTTCATG	135	1.96
*cyp2p7*	BX081233	CAGGAGGGCAAGTTCAGGAA	CAGCAGGGAGGTGAAGAACAG	117	2.03
*cyp3a27*	U96077	TGCTCAAGTTCGACCTGTTCA	CCAGCGAGGCGTAAAAGAAG	132	1.99
*hmox1*	BX317345	CACGCCTACACCCGTTACCT	AAGCGGTTAGGGCTTGACACT	137	1.91
*igfbp1*	NM_001124561	GGGTATACACGGCCCACTGT	CCCTGGTCCTCAGTGCAGAT	112	1.88
*rxra*	EU084731	TCCATCGGCCTCAAGTGTCT	AGGAACGTGTCTATGGGCGTAT	74	2.02
*mta*	CR944007	ATCTTGCAACTGCGGTGGAT	GCAGCCTGAGGCACACTTG	115	1.97
*mtb*	HQ142689	CCGTCTGTCCCTGACGCTAT	CATTTTTCGATCGCGCTTCT	120	1.96
*cat*	CA381929	GCATCCCAGAGAGGGTGGTA	GTCTTGCCCACATGCTCAAA	115	2.03
*mnsod*	CA375800	GGCCATCAACCGTGACTTTG	AGCTTCCCGCTCTCCTTGTC	120	2.04
*tf*	NM_001124552	CGGCTATACCGGAGCATTCA	TTCAGGTCCTGTGCCCAAAC	120	1.99
*ppara*	NM_001197211	ACAATGCGATCCGTTTTGGT	TGCCTGGCCAAAGTCTTCTG	124	1.99
*alb*		GACTACGACCCCTCCAGCATT	GGGCATGAACTCCGTGTCA	111	2.01
*chf*	NM_001124427	GCCAATGGCCACTACAGCAT	GCTCCATCCTGCCACCATAC	132	2.01
*timp2*	NM_001124508	CAGCCGTTTGTGGCGTTACT	TGGCCTCCCAAGACTCAATAA	123	2.08
*lxr*	NM_001145421	ACCTGGACGAGGCGGAGTAT	GAACGAAGTGCGTCCACGTA	124	2.05
*slc27a2*	CB497596	GTGTTCAAGTACCAGGCAACGA	GCATACGCCGGTAGGTAGCT	121	2.01
*actb*	NM_001124235	GTTGGGATGGGCCAGAAAG	TCGTCCCAGTTGGTGACGAT	110	1.87
*rpl13*	NM_001160671	GTGTCCCCACTTGGCTGAAG	CCGTGGGAGTCCCTTAGAATG	115	1.93
*ubi*	NM_001124194	GAGGATGGTCGCACCTTGTC	CTGGGCCAGCTGTCTCAAAG	111	2.02

RT-qPCR was conducted on the RNA isolated from the 15 individual liver samples as previously described by Olsvik et al. [16]. Briefly, a two-step real-time RT-PCR protocol was used to quantify the transcriptional levels of the selected genes. The RT reactions were run in duplicate on a 96-well reaction plate with the GeneAmp PCR 9700 machine (Applied Biosystems, Foster City, CA, USA) using TaqMan Reverse Transcription Reagent containing Multiscribe Reverse Transcriptase (50 U µL^−^) (Applied Biosystems, Foster City, CA, USA). Two-fold serial dilutions of total RNA were made for efficiency calculations. Six serial dilutions (1000–31 ng RNA) in triplicates were analyzed in separate sample wells. Total RNA input was 500 ng in each reaction for all genes. No template controls (ntc) and RT-controls were run for quality assessment for each PCR assay.

Reverse transcription was performed at 48°C for 60 min by using oligo dT primers (2.5 μM) for all genes in 50 µL total volume. The final concentration of the other chemicals in each RT reaction was: MgCl_2_ (5.5 mM), dNTP (500 mM of each), 10× TaqMan RT buffer (1X), RNase inhibitor (0.4 U µL^−^) and Multiscribe reverse transcriptase (1.67 U μL) (Applied Biosystems). Twofold diluted cDNA (2.0 μL cDNA in each RT reaction) was transferred to 384-well reaction plates and the qPCR run in 10 μL reactions on the LightCycler 480 Real-Time PCR System (Roche Applied Sciences, Basel, Switzerland). Real-time PCR was performed using SYBR Green Master Mix (LightCycler 480 SYBR Green master mix kit, Roche Applied Sciences,), which contains FastStart DNA polymerase, and gene-specific primers (500 nM of each). PCR was achieved with a 5 min activation and denaturizing step at 95°C, followed by 45 cycles of a 10 s denaturing step at 95°C, a 20 s annealing step at 60°C and a 30 s synthesis step at 72°C. Target gene mean normalized expression (MNE) was determined using a normalization factor based upon *actb*, *rpl13* and *ubiquitin*, as calculated by the *geNorm* software [17]. All these transcripts were stably expressed among the 30 evaluated samples, with *geNorm* stability scores of M<0.28.

### 2.7. Statistics

Statistical treatment of the biological data was performed using the program Statistica 8.0; 2008 (Statsoft Inc., Tulsa, USA). Significant differences among dietary treatments were assessed by a one-way analysis of variance (ANOVA) using tank as replication unit (n = 3 per dietary treatment). *Post hoc* testing of significant differences was assessed by using a Tukey's HSD test. The GraphPad Prism 5.0 software (GraphPad Software, Inc., San Diego, CA, USA) was used for statistical analyses of the transcriptional data. T-test was used to compare the transcriptional levels of the examined genes between the two experimental groups. Contigs and isotigs were annotated with the Blast2GO software. The functional pathway analyses were generated through the use of IPA (Ingenuity Systems, www.ingenuity.com). A significance level of P<0.05 was used for all tests.

## Results

### 3.1. Feeding trial and growth

Table 1 show the experimental basal feed recipe, while Table 2 includes target supplementations of vitamins and minerals from the premix in the unsupplementary Diet U and supplementary Diet S. The feed levels of micronutrients in the Diet S reflected the supplementation from the premix (Table 2), however, different efficiency was obtained depending on nutrient stability (Table 2). Of the vitamins, vitamin K_3_ showed largest feed losses (10% of added), followed by vitamin B_12_ (28%), folic acid (46%), pyridoxine (54%), vitamin E (59%), biotin (68%), thiamine (71%), pantothenic acid (73%), riboflavin (75%), ascorbic acid (82%) and niacin (106%). The element retention of supplemented were all above 80%.

The biological material in this study was collected from the final sampling of a 10 weeks feeding experiment with a minor phenotypic change in growth pattern that could suspect an initial suboptimal micronutrient supply in fish fed Diet U. The fish grew well during the 10 weeks (from 28 g to 158 g; SGR; 2.56±0.04% day^−1^), with a 5.6 times relative increase in body weight. Dividing the feeding experiment into 5 weeks intervals indicated no difference in the first period (Diet U: 3.07±0.03% day^−1^; Diet S: 3.08±0.05% day^−1^; n = 3), while a reduced specific growth rate were observed in the second period for fish fed the diet without premix supplementation (Diet U: 1.91±0.08% day^−1^; Diet S: 2.08±0.03% day^−1^; n = 3; p<0.05). Mortality was neglectable in both feeding groups.

The study included analyses of nutrient status parameters in the rainbow trout fed Diets U and S (Table 2). Among these measures showing nutrient ratio (Diet S/Diet U) in descending order, liver vitamin C (8.5), liver vitamin E (3.9), muscle pantothenic acid (3.3), muscle pyridoxine (2.9), whole body zinc (1.6) and liver folic acid (1.4) increased significantly in status in fish fed diet S relative to fish fed diet U (Table 2). The other micronutrients did not differ among the groups and was therefore considered sufficient in fish fed Diet U without premix supplementation (Table 2).

**Figure 1:**
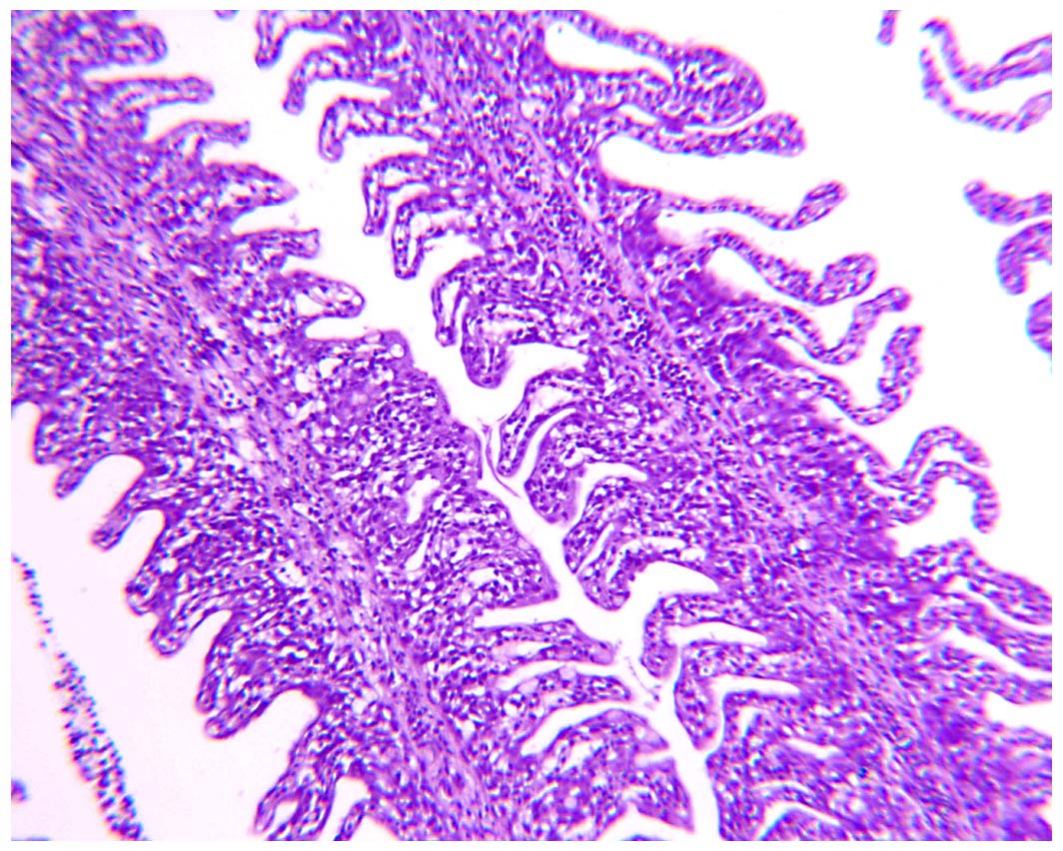
Gill histopathology observed epithelial hyperplasia in rainbow trout fed Diet U without vitamin and mineral premix for 10 weeks, as compared to normally appearing gills in fish fed the supplemented Diet S. (Photos: Dr. Anne-Berit Olsen, Norwegian Veterinary Institute, Bergen, Norway).

Gill histology performed on fish fed Diet U and Diet S from the final sampling showed mainly pathological changes in fish fed the unsupplemented diet, indicating a marginal micronutrient supply compared fish fed a complete diet. The histopathological changes included mononuclear infiltrations and epithelial hyperplasia (Figure 1). Some of the control fish fed Diet S showed minor bleedings/teleangiextasia, probably related to the sampling.

### 3.2. 454 FLX sequencing

A total of 552 812 reads were sequenced using 454 FLX GS Titanium technology from two SSH cDNA libraries. 262 064 reads were sequenced from the forward library made from RNA obtained from liver of rainbow trout fed the unsupplemented diet (Diet U), and 301 000 reads were sequenced from the reverse library made from RNA obtained from liver of rainbow trout fed the supplemented diet (Diet S). Reads were assembled with Newbler software either separately from each cDNA library, or in an overall super-assembly, which included reads from both cDNA libraries. Using Newbler (GS De Novo Assembler, v2.6.), 79.7% of the reads were included in the super-assembly, and of these 70.8% were assembled. The assembly yielded 6 671 isotigs with an average size of 498 bases, and 8 956 contigs and 86 288 singletons.

To search for possible impurities in the cDNA pool, a potential issue with 454 sequencing, MEGAN analyses were done. Metatranscriptomic analysis showed that reads from both cDNA libraries had very few hits against microbial sequences, less than 0.3% in both libraries, and with a vast majority of hits against sequences from teleostean species, clearly suggesting a low level of contamination in the cDNA pools. A surprisingly large number of reads gave no hits against GenBank entries using a BLASTX e-value cutoff of 10^−6^. For the forward SSH cDNA library there were about 48% reads with no hits, while for the reverse SSH cDNA library there were 49% reads with no hits.

A GAFFA (Genome Annotation Framework for Flexible Analysis) web-based project database, developed at the Computational Biology Unit (CBU), University of Bergen, was established as a framework for analysis, management, integration and storage of the genomics data. For any database request, please contact the corresponding author.

### 3.3. Annotation and gene ontology (GO) enrichment analysis

**Figure 2:**
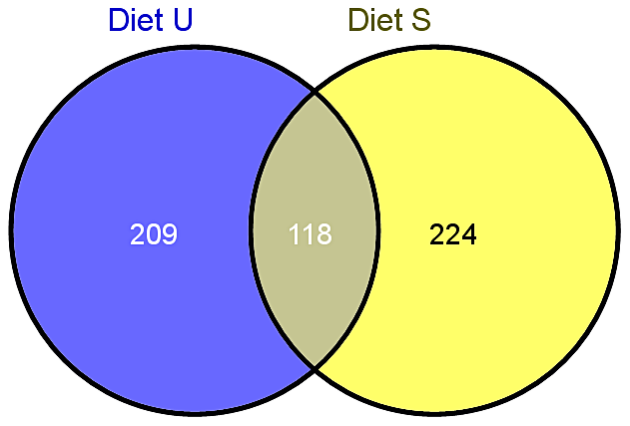
Number of transcripts significantly different expressed in rainbow trout fed Diet U and S. Venn diagram [48]. Based on isotigs assembled from more than 50 read with human IDs (IPA readable). Diet U: 209 uniquely mapped transcripts from a total of 448 molecules. Diet S: 224 uniquely mapped transcripts from a total of 533 molecules.

For downstream functional analyses, assembled isotigs from the two SSH cDNA libraries were used. To limit the number of sequences to be analyzed, only assembled isotigs consisting of more than 50 reads obtained from each of the two libraries were used for downstream analysis. This provided 636 sequences as input for Blast2GO analysis from the Diet U library and 662 sequences from the Diet S library. Annotations were obtained by running Blast2GO with a BlastX cutoff of 10^−6^. 420 isotigs from the Diet U library and 501 isotigs from the Diet S library were annotated using Blast2GO software. Tables S1 and S2 lists these 921 isotig sequences from the Diet U and Diet S libraries, including sequence names, length and descriptions, with annotations as of February 2012. Figure 2 shows numbers of unique annotated sequences in the Diet U and Diet S libraries, and common sequences annotated to the same gene between the two libraries in a Venn diagram.

GO enrichment analysis was performed by using the Fisher Exact Test, as implemented in the Blast2GO software, to study dietary-specific responses in rainbow trout using isotigs containing more than 50 reads, sequenced from the SSH libraries Diet U and Diet S. Fisher's Exact Test was used to assess the significance of such differences. Table 4 shows the GO IDs and terms, P-values, and the number of transcripts associated with a specific GO term from the two libraries. GO enrichment analysis found that 30 GO terms were differentially represented between the Diet U and Diet S libraries, with 9 being overrepresented and 21 underrepresented in rainbow trout fed Diet U. Several overrepresented terms were associated with *lipid metabolic processes* and *ATPase activity*, whereas several *hematological processes* were underrepresented. The GO enrichment analysis also suggests that growth was suppressed in fish fed the unsupplementary diet (Diet U), supported by a trend of growth depression.

**Table 4:** Differential Gene Ontology terms between contigs derived from Diet U and Diet S SSH cDNA libraries.

GO ID	GO Term	Category	P-Value	Diet U	Diet S
GO:0006629	lipid metabolic process	Up	0,004	25	12
GO:0009611	response to wounding	Down	0,006	14	40
GO:0016887	ATPase activity	Up	0,007	12	3
GO:0006643	membrane lipid metabolic process	Up	0,007	6	0
GO:0050817	coagulation	Down	0,009	10	31
GO:0042623	ATPase activity, coupled	Up	0,012	11	3
GO:0006631	fatty acid metabolic process	Up	0,013	8	1
GO:0050878	regulation of body fluid levels	Down	0,014	10	30
GO:0042060	wound healing	Down	0,014	10	30
GO:0009792	embryonic development ending in birth or egg hatching	Down	0,016	1	11
GO:0043009	chordate embryonic development	Down	0,016	1	11
GO:0006664	glycolipid metabolic process	Up	0,016	5	0
GO:0006665	sphingolipid metabolic process	Up	0,016	5	0
GO:0006687	glycosphingolipid metabolic process	Up	0,016	5	0
GO:0005976	polysaccharide metabolic process	Down	0,017	3	15
GO:0007596	blood coagulation	Down	0,020	10	29
GO:0007599	hemostasis	Down	0,020	10	29
GO:0019825	oxygen binding	Down	0,020	0	7
GO:0031406	carboxylic acid binding	Down	0,020	0	7
GO:0015669	gas transport	Down	0,020	0	7
GO:0015671	oxygen transport	Down	0,020	0	7
GO:0005344	oxygen transporter activity	Down	0,020	0	7
GO:0008236	serine-type peptidase activity	Down	0,028	12	31
GO:0017171	serine hydrolase activity	Down	0,028	12	31
GO:0008233	peptidase activity	Down	0,033	23	49
GO:0044255	cellular lipid metabolic process	Up	0,035	16	8
GO:0020037	heme binding	Down	0,035	9	26
GO:0048589	developmental growth	Down	0,037	0	6
GO:0005515	protein binding	Down	0,041	53	93
GO:0040007	growth	Down	0,046	2	11

GO enrichment analysis between sequences generated form Diet U (forward subtracted library from the fish fed the unsupplementary diet) and Diet S (reverse subtracted library from the from the fish fed the supplementary diet) SSH libraries using Fisher's exact test with p≤0.05. Category Red: up-regulated in Diet U, Green: down-regulated in Diet U. The numbers of transcripts associated with a specific GO term in their respective libraries is provided.

### 3.4. Functional analyses

IPA-Core analysis was used for evaluation of biological processes, pathways and networks. In order to use IPA, all identifiers must be recognized as mammalian homologs. Some fish-specific genes obviously cannot be given human ortholog names recognized by IPA, and thus cannot be included in the IPA-Core analysis. For example, genes such as fish virus induced trim protein, type-4 ice-structuring protein, antifreeze protein 4 (AFP4) or c type lectin receptor from the gene lists were not included in the IPA functional analyses. Using the gene lists presented in Tables S1 and S2, IPA could map 380 isotig sequences for functional analysis from the Diet U library and 454 isotig sequences from the Diet S library.

**Figure 3:**
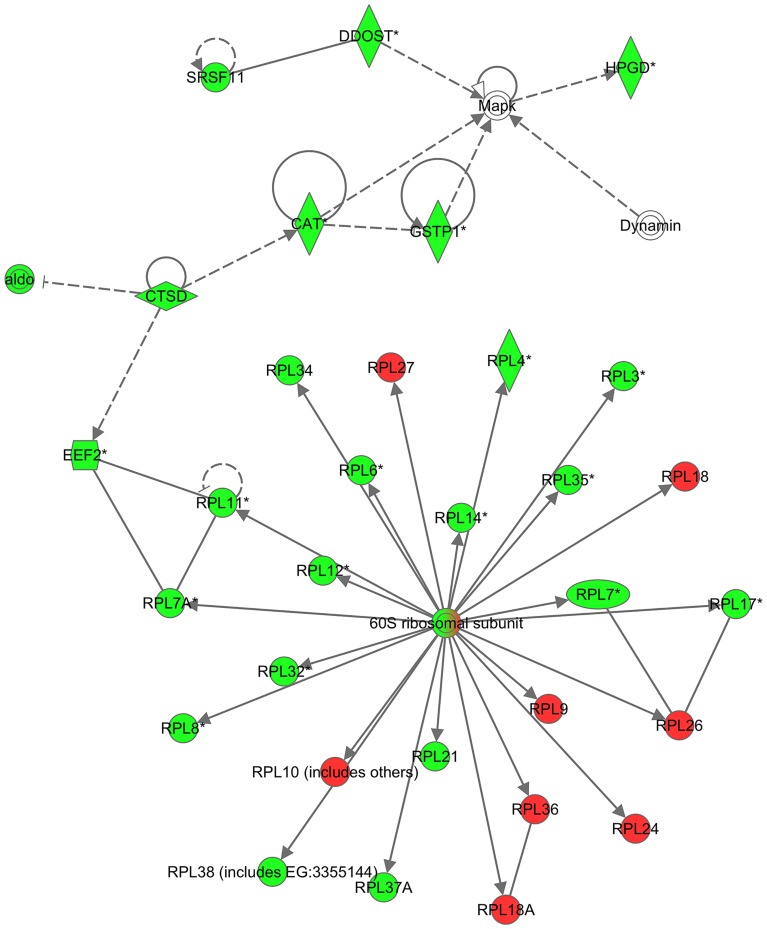
The highest scored network in Ingenuity Pathway Analysis (IPA) in liver of rainbow trout fed the unsupplementary vitamin and mineral Diet U. Arrows indicate direct (solid) and indirect (dashed) relation between molecules. The red color indicates up regulation and green indicates down regulation. The colorless molecules are molecules added by IPA for network generation and are not among the submitted genes from next generation sequencing and RT-qPCR data.

According to the IPA-Core analysis based on transcripts expressed in each of the two cDNA libraries, the top five networks (with 70 molecules per network) and scores from the Diet U library were “*Cell Death, Protein Synthesis, Antigen Presentation*” (score 40), “*Tissue Morphology, Cancer, Connective Tissue Development and Function*” (score 40), “*Cell-To-Cell Signaling and Interaction, Cellular Growth and Proliferation, Hematological System Development and Function*” (score 38), “*Amino Acid Metabolism, Drug Metabolism, Molecular Transport*” (score 37), and “*Cellular Growth and Proliferation, Cell Death, Cellular Movement*” (score 27). The corresponding top five networks from the Diet S group were “*Cell Morphology, Cellular Assembly and Organization, Nervous System Development and Function*” (score 54), “*Cell-To-Cell Signaling and Interaction, Tissue Development, Cellular Compromise*” (score 38), “*Small Molecule Biochemistry, Drug Metabolism, Lipid Metabolism*” (score 36), “*Protein Synthesis, Energy Production, Lipid Metabolism*” (score 36), and “*Cell-To-Cell Signaling and Interaction, Tissue Development, Hematological System Development and Function*” (score 29). By defining all sequences enriched in the forward library (Diet U) as being up-regulated in the Diet U group and given a score 2, and by defining all sequences enriched in the reverse library (Diet S) as being down-regulated in the Diet U group with a score -2, IPA-Core analysis was performed with all mapped molecules in the two libraries. Using this approach, the top five IPA-Core analysis networks were “*Protein Synthesis, Gene Expression, RNA Post-Transcriptional Modification*” (score 93), “*Cellular Assembly and Organization, Hepatocellular Peroxisome Proliferation, Reproductive System Disease*” (score 68), “*Developmental Disorder, Genetic Disorder, Metabolic Disease*” (score 65), “*DNA Replication, Recombination, and Repair, Energy Production, Nucleic Acid Metabolism*” (score 59) and “*Lipid Metabolism, Small Molecule Biochemistry, Endocrine System Development and Function*” (score 56). Figure 3 shows the top network, set with a 35-molecule limit, focusing on protein synthesis. Two IPA Core analysis Top Bio Pathways gave a significant z-score, a regulation score measuring consistency of a gene expression pattern, for two pathways. These were “*Synthesis of fatty acids*” (22 molecules, P = 7.85E^−5^, z-score = 2.33) and “*Hydrolysis of protein*” (13 molecules, P = 2.24E^−3^, z-score = 2.00).

The IPA-Compare function was used to analyze molecules unique to either of the Diet U and Diet S fish groups. Associated function of the unique top canonical pathways in the Diet U group was (with scores in parentheses) “*EIF2 Signaling*” (P-value 1.95E-14, ratio 19/205 (0.093)), “*Oxidative Phosphorylation*” (P-value 1.71E-12, ratio 16/159 (0.101)), “*Mitochondrial Dysfunction*” (P-value 2.49E-07, ratio 11/174 (0.063)), “*Regulation of eIF4 and p70S6K Signaling*” (P-value 1.47E-05, ratio 9/176 (0.051)), and “*LXR/RXR Activation*” (P-value 2.56E-05, ratio 8/136 (0.059)). The corresponding unique top five canonical pathways for the Diet S fish group were “*EIF2 Signaling*” (P-value 1.13E-15, ratio 21/205 (0.102)), “*Regulation of eIF4 and p70S6K Signaling*” (P-value 7.21E-07, ratio 11/176 (0.062)), “*mTOR Signaling*” (P-value 8.45E-06, ratio 11/211 (0.052)), “*Acute Phase Response Signaling*” (P-value 7.83E-05, ratio 9/177 (0.051)), and “*PXR/RXR Activation*” (P-value 1.15E-04, ratio 6/88 (0.068)).

The top tox list (IPA-Tox) from this analysis is shown in Table 5 (Diet U, Diet S and combined), including ratios of the five most significant pathways. According to this analysis the unsupplementary Diet U mediated mitochondrial dysfunction and effects on lipid metabolism, while the supplementary Diet S mediated effects on genes encoding proteins involved in detoxification and oxidative stress.

**Table 5:** IPA-Tox lists with significant responses in rainbow trout fed Diet U or Diet S.

Group	Name	p-value	Ratio	Group	Name	p-value	Ratio
Diet U	Mitochondrial Dysfunction	5.31E-06	9/138 (0.065)	Diet S	PXR/RXR Activation	6.92E-05	6/69 (0,087)
Diet U	LXR/RXR Activation	1.16E-04	7/119 (0,059)	Diet S	Cytochrome P450 Panel – Substrate is a Xenobiotic (Human)	7.39E-04	3/18 (0,167)
Diet U	Fatty Acid Metabolism	9.7E-04	6/123 (0,049)	Diet S	Cytochrome P450 Panel – Substrate is a Xenobiotic (Mouse)	1.98E-03	3/25 (0,12)
Diet U	Negative Acute Phase Response Proteins	2.27E-03	2/8 (0,25)	Diet S	Oxidative Stress	2.62E-03	4/57 (0,07)
Diet U	LPS/IL-1 Mediated Inhibition of RXR Function	6.29E-03	7/236 (0.03)	Diet S	Decreases Depolarization of Mitochondria and Mitochondrial Membrane	3.48E-03	2/9 (0,222)
Common Diet U and S	Positive Acute Phase Response Proteins	3.2E-15	14/30 (0,467)				
Common Diet U and S	LXR/RXR Activation	1.58E-11	20/119 (0,168)				
Common Diet U and S	Negative Acute Phase Response Proteins	4.64E-11	7/8 (0,875)				
Common Diet U and S	Mitochondrial Dysfunction	2,00E-06	15/138 (0,109)				
	PXR/RXR Activation	8.02E-06	10/69 (0,145)				

For the Common Diet U and S group, transcripts enriched in the forward (Diet U and reverse (Diet S) libraries were given a positive or negative fold change score, respectively. Based on the transcripts expressed in liver of rainbow trout from the two dietary groups or common for the two groups.

Table 6 shows vitamin and mineral metabolism relevant gene transcripts affected by the two experimental diets in liver of rainbow trout, as determined by IPA core analysis Top Bio Functions (molecular and cellular functions). Annotation function, p-values, and individually affected genes, including total number of genes, are shown. Metabolism of terpenoid scored highest, with a p-value of 10.31E-10 and 32 molecules included in the dataset.

**Table 6:** Vitamin and mineral metabolism relevant gene transcripts affected by the two experimental diets in liver of rainbow trout.

Category	Functions Annotation	p-Value	Molecules	# Molecules
Vitamin and Mineral Metabolism	metabolism of terpenoid	3.51E-10	ADH1C,ADH5(includesEG :11532),ANGPTL3,APOA1,APOA2, APOA4,APOB,ATP,CAT,CYP2B6, CYP2C8,CYP2G1P,CYP2J2,CYP3A4, CYP51A1, CYP7A1,CYP8B1,G6PC, G6PD,HNF1A,HSPA8,NADPH,NR0B2, PLG,RBP1,RBP2,RDH5,SERPINA1, SLC37A4,SULT1A1,TSPO,TTR	32
Vitamin and Mineral Metabolism	steroid metabolism	6.08E-07	ANGPTL3,APOA1,APOA2, APOA4,APOB,ATP,CAT,CYP2B6, CYP2G1P,CYP3A4,CYP51A1,CYP7A1, CYP8B1,G6PC,G6PD,HNF1A,HSPA8,NADPH, NR0B2,PLG,SERPINA1,SLC37A4,SULT1A1,TSPO	24
Vitamin and Mineral Metabolism	Metabolism of retinoid	1.55E-06	ADH1C,ADH5 (includes EG:11532),CYP2B6,CYP2J2, CYP3A4,NADPH,RBP1, RBP2,RDH5,TTR	10
Vitamin and Mineral Metabolism	Metabolism of cholesterol	2.92E-06	ANGPTL3,APOA1,APOA2, APOA4,APOB,CAT,CYP51A1,CYP7A1, CYP8B1,G6PD,HNF1A,NR0B2, SERPINA1	13
Vitamin and Mineral Metabolism	Metabolism of paclitaxel	1.85E-05	CYP2B6,CYP2C8,CYP3A4	3
Vitamin and Mineral Metabolism	Metabolism of vitamin	2.04E-05	ADH1C,ADH5 (includes EG:11532),CYP2B6,CYP2J2,CYP3A4, HSPA8,NADPH,RBP1,RBP2,RDH5,TTR	11
Vitamin and Mineral Metabolism	Metabolism of tretinoin	1.69E-04	ADH1C,ADH5 (includes EG:11532),CYP2B6,CYP3A4, NADPH,RBP1	6
Vitamin and Mineral Metabolism	Homeostasis of cholesterol	1.94E-04	ANGPTL3,APOA1,APOA2,APOA4, APOB,CYP7A1,FN1,G6PC,SLC37A4	9
Vitamin and Mineral Metabolism	Metabolism of retinol	6.37E-04	ADH1C,CYP3A4,RBP1,RBP2,TTR	5
Vitamin and Mineral Metabolism	synthesis of cholesterol	1.01E-03	APOA1,APOB,CYP51A1,CYP7A1,CYP8B1,G6PD,SERPINA1	7
Vitamin and Mineral Metabolism	synthesis of sterol	1.25E-03	APOA1,APOB,CYP51A1,CYP7A1, CYP8B1,G6PD,HSPA8,SERPINA1	8
Vitamin and Mineral Metabolism	synthesis of terpenoid	3.18E-03	ADH1C,ADH5 (includes EG:11532),APOA1,APOB,ATP,CYP51A1, CYP7A1,CYP8B1,ESR2,G6PD,HNF1A,HSPA8, NADPH,PLG,RDH5,SERPINA1,TSPO	17
Vitamin and Mineral Metabolism	concentration of tretinoin	4.06E-03	ADH1C,RBP1	2
Vitamin and Mineral Metabolism	metabolism of testosterone	4.06E-03	CYP2G1P,CYP3A4	2

IPA core analysis top bio functions (molecular and cellular functions).

IPA Transcription Factor Analysis predicts which transcription factors are activated or inhibited to explain the observed gene expression changes in the experimental dataset. Its underlying method is based on two complementary statistical quantities, an overlap p-value measuring significant overlap between genes regulated by a transcription factor and dys-regulated genes in the data set, and a regulation z-score measuring consistency of a gene expression pattern with the activation or inhibition of a given transcription factor. In rainbow trout fed the unsupplemented diet (diet U) estrogen-related receptor alpha (ESRRA) and forkhead box A1 (FOXA1) were predicted activated with a regulation z-score of 2.7 and 2.4, respectively. CREB binding protein (CREBBP) and hepatocyte nuclear factor 4 alpha (HNF4A) were predicted inhibited with z-scores of −2.2 and −2.6.

### 3.5. RT-qPCR analyses

**Figure 4:**
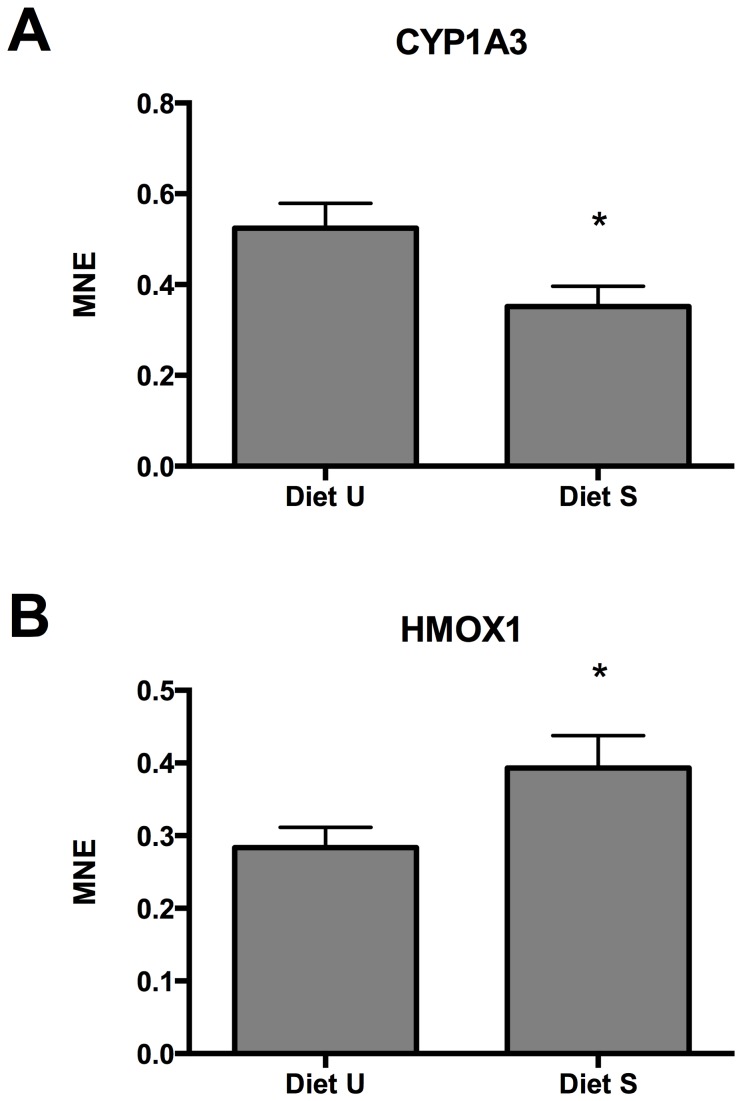
Significantly different expression of A) *cyp1a3*, and B) *hmox1* in liver of rainbow trout fed diets with unsupplemented (Diet U) or supplemented (Diet S) levels of a vitamin and mineral premix. (Mean ± SEM). * P<0.05.

Based on the findings from the SSH cDNA libraries, the transcriptional levels of a set of genes were determined with RT-qPCR from a total of 30 individual rainbow trout (15 fish from each dietary group). These genes were selected as potential markers of pathways suggested affected by the SSH cDNA libraries or as expected candidate biomarkers based on current knowledge. Table 3 lists the target genes selected for RT-qPCR verification. Only two out of 17 evaluated transcripts were significantly different between the two dietary groups (t-test). These were *cyp1a3* (Figure 4A), and *hmox1* (Figure 4B). We do at present not know why so few of these transcripts, especially the ones selected from the cDNA library lists, turned out not to be significant differentially regulated between the two fish groups according to the RT-qPCR analyses. The reason for the observed discrepancy for some of the transcripts may rely on a number of inherent aspects with two methods, e.g. normalization procedures. Spearman rank correlation was used to search for possible co-variation in transcriptional levels of the 17 targets genes based on the RT-qPCR data, and for possible size-dependent transcriptional tendencies. No strong correlations were observed among the 17 studied target genes. Neither did any of the analyzed transcripts grouped distinctly together with one of the two dietary groups according to the Principal Component Analysis (PCA).

## Discussion

The biological performance was seemingly not different between the two diets in the present study, with just a minor growth depression in rainbow trout fed the unsupplemented diet during the second half of the experiment. For most of the analyzed feed nutrient levels, lower levels than supplemented were found for Diet S, most probably due to losses during feed production [18], [19]. Of the analyzed corresponding biological status markers, fish responded to the antioxidant vitamins C and E, the metabolic cofactors pantothenic acid and pyridoxine, and developmental factors folate and zinc in a decreasing order of magnitude.

The GO enrichment analysis suggested that growth was suppressed in rainbow trout fed the Diet U. By dividing the experimental periods into two five weeks intervals, a mild growth depression in fish fed the unsupplemented diet was seen during the last period relative to fish fed the supplemented diet [11], supporting the gene analysis. This can be interpreted as an initial state of micronutrient deficiency, most probably related to lack of pantothenic acid. Similarly, Matsumoto et al. [20] showed pantothenic acid deficiency related reduction in feed intakes and growth in 150 g rainbow trout after doubling its weight on an unsupplemented semi-purified diet for ∼20 weeks. In the present study muscle pantothenic acid indicated a state of deficiency in fish fed Diet U, with 2.3 times increase in concentration when the diet was supplemented micronutrient premix. In the study by Masumoto et al. [20] similar difference in gill pantothenic acid status was seen, while liver pantothenic acid only differed ∼40%. Clubbed gills is the most characteristic deficiency sign in salmonids at pantothenic acid deficiency [2], [3], [21]. The results from the present feeding study support that muscle and gills were impacted in the unsupplemented group, as indicated by an initial growth reduction in the last five weeks and early histopathological changes in the gills after ten weeks.

Coenzyme A is the active coenzyme form of pantothenic acid, which participate in over 70 acyl transfer reactions in energy production (acetyl CoA), fatty acid oxidation, cholesterol synthesis, heme synthesis, amino acid catabolism, acetylcholine synthesis, and other less well known acetylation and acylation reactions. Thus, mitochondria-rich tissues, like the gills, are very sensitive to states of deficiency. The GO enrichment analysis identified overrepresented terms associated with *lipid metabolic processes* and *ATPase activity*, whereas several *hematological processes* were underrepresented. From Table 2, pantothenic acid was among the micronutrients in Diet U (6 mg kg^−1^) that was below the established requirement for rainbow trout (20 mg kg^−1^), and among the most responding micronutrients in trout fed the Diet S. Thus, with an initial suboptimal pantothenic acid status, it is likely that lipid oxidation, ATP generation and heme synthesis are affected, even though liver seems to be a prioritized tissue for this vitamin with less difference in status between states of sufficiency and deficiency [20]. The high numbers of up-regulated transcripts associated with Diet U (Table 4) can interpreted as initiation of compensatory metabolic mechanisms for energy generation at suboptimal pantothenic acid status. Similarly, the IPA-Tox lists of Diet U, Diet S and their combined, included ratios of the five most significantly affected pathways. At top of the list of Diet U ranges mitochondrial dysfunction, activation of liver lipid and cholesterol metabolism and transportation, sensed through the liver X receptor LXR/RXR system. The latter is valid also for Diet S and the common list for Diets U and S, underlining an alarmed signaling system at gene transcription level in early nutrient (here mainly pantothenic acid) deficiency, since neither whole body lipid retention, liver lipid concentration nor blood lipids differed between the groups U and S (own unpublished data).

In more details, the 14 functional annotations identified by the IPA core analysis can be divided into synthesis, metabolism and homeostasis of terpenoids, steroids (testosterone), vitamins (retinoids, retinol, retinoic acid or tretinoin) and cholesterol (sterols) (Table 6). Terpenoids (or isoprenoids) are one of the largest classes of organic compounds, characterized by one or more isoprene units. Cholesterol and steroids are synthesized from terpenoid precursors, which in turn are synthesized through the mevalonate pathway, starting with formation of acetoacetyl CoA from two molecules acetyl CoA. Clearly, pantothenic acid status can be linked to most of these functions annotations, which can serve as functional gene markers for suboptimal pantothenic acid nutrition. From the IPA core analysis, paclitaxel metabolism was also affected. Paclitaxel is a plant derived compound inhibiting cell division and is therefore used as cytostatica in human medicine. The compound is however metabolized through the commonly abundant cytochrome P450 enzymes in the mammalian liver (CYP3A4, CYP2B6, CYP2C8) [22], also involved in metabolism of large number of substrates like xenobiotics and terpenoids, and in synthesis of cholesterol, steroids and other lipids (CYP2B6). It is therefore tempting to associate this mammalian homolog identifier to the impacted lipid synthesis and metabolism functions, as discussed above. In line with this, upregulated transcription of CYP1A3, as part for the cytochrome P450 panel was verified with RT-qPCR in the individual trout (n = 15) fed diet without micronutrient supplementation compared to fish fed the supplemented diet (Figure 4; Table 5).

Since bony fish, like the salmonids are not able to synthesize vitamin C or ascorbic acid [23], vitamin C deficiency has been one of the most serious causes for mortalities in the aquaculture nutrition history, with symptoms as poor performance, anemia, bone and skin disorders, and increased susceptibility to infections [24], [25]. Suboptimal dietary vitamin C status in larger fish is however not easily detected. Compared to liver status in Atlantic salmon, *Salmo salar* [26], [27] and to status of the rainbow trout fed diet S (93 µg g^−1^) supplemented ascorbic acid phosphate, liver ascorbic acid status in the trout fed Diet U (11 µg g^−1^) was borderline deficient. Suboptimal vitamin C status can be expected to affect roles such as collagen structure, wound repair, mineral metabolism (especially iron and copper), hematology, stress response, immunity, and detoxification reactions (reviewed by [24], [25], [28]). Vitamin C is a multifunctional vitamin, acting as cofactor for a multitude of enzymatic oxidation and hydroxylation reactions (posttranslational modification of amino acids in collagen, steroidogenesis, detoxification among others), as well as antioxidant supporting iron metabolism and hemoglobin synthesis by keeping iron in a reduced state. Many of the down-regulated differential GO terms identified in Table 4 could therefore be ascribed suboptimal vitamin C status, such as wound healing, hematology, oxygen transport, embryonic development, tissue development and growth. RNA expression of heme oxygenase (*hmox1*), catalyzing degradation of heme, was down regulated in fish fed Diet U (Figure 4). Arguments for protecting the heme from degradation in these fish are evident, among others due to the deficient CoA-acyl transportation and mitochondrial dysfunction and lack of reducing capacity of ascorbic acid to mobilize iron for heme synthesis [29].

It is generally accepted that micronutrients must be present at optimal levels to ensure proper immune function in animals and humans. The significance of deficiency and excess of dietary ascorbic acid for fish innate and specific immunity has been extensively focused (see [9], [25], [30], among others related the mechanisms to stress alleviating effects, increased activity of innate factors like complement and lysozyme activities, steroidogenesis, mixed function oxidases and antioxidation. Possibly linked to differential ascorbic acid status, the IPA-Tox lists identified negative acute phase response proteins and negative regulative immune activities affected for Diet U fish (Table 5), while activation of the LXR/RXR and cytochrome P450, and oxidative stress activities for the Diet S fish. In scrurvy-prone rats, hepatic acute phase response expression was increased at vitamin C deficiency, suggesting that ascorbic acid deficiency causes physiologic changes similar to those that occur in an acute phase response [31]. Besides supporting cholesterol metabolism and reducing cellular oxidative stress in fish fed Diet S, it is assumed that excess vitamin and mineral supplementation through the premix activated the cytochrome P450 systems through an increased load of organic and potential oxidative elements. As the first barrier towards excess dietary input, the intestine effectively regulates uptake of elements [30], [32]. Digestibility of the supplemented elements in the present diets was considerably higher in Diets U than Diet S (own unpublished data), indicating that the fish in the Diet S group down-regulated the intestinal uptake of excess elements, possibly also affecting the liver metabolism and protecting mechanisms.

Vitamin E concentration in Diet U was sufficient according to NRC [2], with liver status of 45 µg α-tocopherol g^−1^, indicating no immediate vitamin E deficiency compared to similar sized Atlantic salmon showing deficiency signs at <10 µg α-tocopherol g^−1^ liver [33]. In addition, plant ingredients contain considerable levels of γ-tocopherol [34], supplying both experimental diets with 44 µg γ-tocopherol g^−1^. Vitamin E deficiency is characterized by decreased concentration of hemoglobin and accumulation of lipid oxidation products in the liver [35], while these parameters were not affected in the present experiment (data not shown). On the other hand, excess vitamin E has been associated with improved innate and acquired immunity [36], possibly through the function as a lipid soluble antioxidant or specific modulator of cell signaling through regulation of enzyme activities and gene expression [37]. No dependence on long-term vitamin E feeding and differential vitamin E status were, however, observed on measured liver transcripts of genes involved in oxidative stress response, lipid metabolism, vitamin E trafficking and cell death in zebrafish, *Danio rerio* [38]. Using a functional feed with vitamin E supplementation Tacchi et al. [9] showed reduced liver transcription of immune parameters, interpreted as a freeing and redirection of energy from immunity towards growth.

Activation of the LXR/RXR system means activation of transcriptional factors involved in sterol catabolism and *de novo* synthesis of fatty acids. Besides environmental and developmental effects, Cruz-Garcia et al. [39] suggested that plant based diets with lower cholesterol levels may lower liver LXR expression in Atlantic salmon. The present diets based on equal parts of plant and marine ingredients, were similar except for the vitamin and mineral premix, while sterol and fatty acid metabolism were affected at transcription level in fish fed the Diet U. Although liver cholesterol concentration was not measured in the present study, corresponding muscle cholesterol concentration was similar between the two groups (data not shown). In longer run, a hepatic metabolic conflict may arise in fish fed diet U, with plant diets reducing LXR and pantothenic acid deficiency activating LXR due to reduced lipid catabolic capacity.

Many of the differential GO terms included down-regulation of genes coding for protolytic activities in the Diet U fish, like proteins in extracellular processes as blood coagulation and fibrinolysis, apoptosis, and immunity. Recently, Page and Cera [40] reviewed enzymes in the degradome which normally constitutes 2–4% of the encoded genes in the total genome in an organism, and of which serine-type peptidases typically makes out 25%. Down-regulation of these genes indicates reduced protein turnover with the early growth depression seen, probably as an energy saving action. On the other hand, vitamin B_6_ (pyridoxine) differed significantly in muscle status between the groups (2.9 times). The muscle transamination activity (measured as the enzyme activity of aspartate amino transferase ASAT) showed 4.4 times higher activity in the supplemented group than the deficient group (Table 2). The possibility to fully activate the ASAT enzyme with active coenzyme (pyridoxal phosphate) *in vitro* suggests that the apoprotein synthesis was not affected. Severely reduced vitamin B_6_ status will however contribute to reduced protein turnover, protein synthesis and ATP generation [41].

Besides affecting protein and amino acid metabolism and turnover, vitamin B_6_ modulates the expression of a variety of genes that respond to hormones. Albrektsen et al. [42] showed that vitamin B_6_ modulated estradiol induced vitellogenin synthesis in Atlantic salmon. Gene transcripts within steroid metabolism were significantly affected in the present study. They were, however more linked to lipid metabolism, as well as oxidation and phase II detoxification reactions (Table 6).

FOXA1 is a member of the Forkhead box class of DNA-binding proteins that play important roles in regulating the expression of genes in cell growth, proliferation, differentiation, and longevity, and seems to be responsive to hormonal manipulation. These hepatocyte nuclear factors are transcriptional activators for liver-specific transcripts such as albumin and transthyretin, and are also interacting with chromatin. Reviewing the roles of forkhead proteins, Cirillo and Barton [43] discussed an adult-specific function for the FOXA that regulates specifically the relation between food intake and increased lifespan, with reference to works showing increased lifespan in worms, flies and mammals associated with reduced food intake or dietary restriction. Feed intake for the second half of the present study was declining in rainbow trout fed Diet U [11], which may have triggered reactions that predicted activation of FOXA1 shown the IPA Transcription Factor Analysis.

Dietary Zn from the feed ingredients seems to be sufficient to meet the requirement in trout fed the diet without a vitamin and mineral premix, even with 40% Zn digestibility recorded [11]. The whole body Zn status reflected however the difference in dietary Zn, and this was 1.6 times higher in the supplemented diet group than in the controls fed unsupplemented diet. Classical roles of Zn in fish are through functioning as cofactor in enzymes related to growth, cell membrane structural integrity, protein and carbohydrate and nucleic acid metabolism, antioxidation and protein synthesis by acting in gene transcription factors. The antioxidant function of Zn is both related to radical scavenging through Cu/Zn superoxide dismutase (SOD), by inducing metallothionein (MT) that binds otherwise oxidative metals, and by inducing reactions for cellular protein repair to sustain the biological function of the proteins through other mechanisms than gene transcription [44]. Kucukbay et al. [45] showed that Zn supplementation decreased oxidative stress in rainbow trout. In zebrafish exposed to low (3 μg L^−1^ water and 26 mg kg^−1^ diet) or sufficient (16 μg L^−1^ water and 233 mg kg^−1^ diet) water and feed Zn concentrations, several general mechanisms in gill tissue were affected at deficiency, like down-regulation of developmental processes (26% of the genes in a 16K oligo nucleotide array), cell cycle, cell differentiation, gene regulation, butanoate metabolism, lysine degradation, protein tyrosin phosphatases, nucleobase, nucleoside and nucleotide metabolism, and cellular metabolic processes [46]. Genes among these groupings were associated to disorders like diabetes, bone tissue development and ionocyte proliferation. In farmed salmonids, development of bone deformities has been suggested to be ascribed suboptimal Zn nutrition. Even though feed Zn was sufficient in both groups and body status did not indicate critical Zn deficiency, it is not unlikely that the trout fed diet U responded to a declining status of Zn and reduced growth rate by affecting transcripts related both to Zn uptake, metabolism and growth factors. Higher Zn absorption is found in low Zn diets [2]. Zheng et al. [46] demonstrated the importance of including several time points for a better understanding of the homeostatic control mechanisms adjusting the fish body to changing Zn status.

Liver folate status was 1.4 times higher in rainbow trout fed diet supplemented folic acid than in fish fed the basal diet, however not considered critical low status in fish fed unsupplemented diet (own observations). Further, folate and vitamin B_12_ interact deeply in many one-carbon transfer reactions, and the feed levels and status of vitamin B_12_ did not differ between the groups (Table 2). Besides functioning biochemically in the synthesis of purines and pyrimidines, folate also affects gene transcription through DNA methylation (epigenetic alterations), and genes for DNA repair and for proteins involved one-carbon metabolism. Folate deficiency affects growth and hematopoiesis, as well as gene expression of liver lipid metabolism, via homocysteinemia and disrupted choline metabolism, phenotypically visualized as development of fatty liver (steatosis) in mice [47]. However, neither whole body nor liver lipid concentrations supported such pathology in the present study (own unpublished observations). As discussed also for pantothenic acid, differential GO terms between the dietary groups included lipid metabolic processes and developmental growth, and it is not unlikely that declining folate status may induce early changes in gene expressions as metabolic compensation. Homocystein remethylation can for example be shifted at folate deficiency from folate to betain dependent remethylation, although implying increased betain requirement.

The material in present study, originating from rainbow trout suspected to suffer from an initial micronutrient deficiency due to an initial growth depression, had the potential to discover early micronutrient deficiency by use of transcriptional profiling. We have demonstrated differences in the liver transcriptome in fish fed diets with or without supplementation of a micronutrient premix that were supported by differential micronutrient status in the fish. Generally, suboptimal micronutrient status impacted transcriptional factors related to the cellular metabolism, functions and structures, and by introducing respective compensatory mechanisms. Supplied in excess, nutrients can support and improve cellular function, health and repair, but may also expose the cell to oxidative conditions. In the present study, it was not the intention to fully discriminate the impact of the selected nutrients from the micronutrient premix on the liver transcriptomics, even though single liver mRNA abundances and low nutrient status could be interpreted as initial deficiency symptoms. The results identified however, the most critical micronutrients, pantothenic acid and ascorbic acid, by their impacts on growth, mitochondria function, lipid metabolism, energy generation and tissue repair, and specifically the steroid metabolism.

## Supporting Information

Table S1 Transcripts up-regulated in liver of rainbow trout by diet U.
(XLSX)

Table S2 Transcripts down-regulated in liver of rainbow trout by diet U.
(XLSX)
