# Correction: Exploring Early Micronutrient Deficiencies in Rainbow Trout (*Oncorhynchus mykiss*) by Next-Generation Sequencing Technology - From Black Box to Functional Genomics

**DOI:** 10.1371/journal.pone.0156668

**Published:** 2016-05-25

**Authors:** Pål A. Olsvik, Gro-Ingunn Hemre, Rune Waagbø

There is an error in Table 2. The entire table was erroneously duplicated from Table 1 and does not contain any of the correct information. The publisher apologizes for the error. Please see the corrected Table 2 here.

**Table 2:** Target supplementations of vitamins and minerals (mg kg^-1^) from the premix in the two experimental diets (unsupplemented Diet U and supplemented Diet S), respective analyzed feed values, present nutrient requirements from the NRC (2011), and significant changes in respective tissue status between the dietary groups.

	Supplemented		Analyzed				
Micronutrient Method	Diet U	Diet S	Diet U	Diet S	NRC 2011*)	Analyzed tissue	Difference in body status**)
*Vitamins*							
Vitamin E [49]	0	100	44	103	50	Liver	3.9
Vitamin K3 (MD) [50]	0	10	0.2	1.2	R	Liver	ns***)
Thiamin (B1) [51]	0	10	3	10	1	Muscle	ns
Riboflavin (B2) [52]	0	12	5	14	4	Muscle	ns
Niacin [53]	0	30	54	86	10	Muscle	ns
Pantothenic acid [53]	0	40	6	36	20	Muscle	3.3
Pyridoxine (B6) [41,54]	0	10	3/35	8/153	3	Muscle	2.9/4.4****)
Biotin [53]	0	0.3	0.3	0.5	0.15	Muscle	ns
Folic acid [53]	0	10	1	6	1	Liver	1.4
Vitamin B12 [53]	0	0.01	0.09	0.09	R	Muscle	ns
Vitamin C [55	0	70	0	57	20	Liver	8.5
*Elements*							
Zinc (Zn) [56]	0	100	60	155	15	Whole body	1.6
Iodine (I) [57]	0	0.6	0.9	1.5	1.1	Whole body	ns
Copper (Cu) [56]	0	1	5	6	3	Whole body	ns
Cobolt (Co) [56]	0	1	0.2	1.2	?	Whole body	ns
Manganese (Mn) [56]	0	8	29	36	12	Whole body	ns

*) For rainbow trout; R: required;? not determined

**) Significant differences in organ status in rainbow trout fed Diets S relative to U (p<0.05) given as status Diet S/status Diet U

***) ns not significant difference

****) Muscle ASAT activity (U/g protein)
